# Biofilm application for anaerobic digestion: a systematic review and an industrial scale case

**DOI:** 10.1186/s13068-024-02592-4

**Published:** 2024-12-18

**Authors:** Getachew Birhanu Abera, Erik Trømborg, Linn Solli, Juline M. Walter, Radziah Wahid, Espen Govasmark, Svein Jarle Horn, Nabin Aryal, Lu Feng

**Affiliations:** 1 Faculty of Environmental Science and Natural Resource Management, Norwegian University of Life Sciences (NMBU), Postbox 5003, 1432 Ås, Norway; 2 Wondo Genet College of Forestry and Natural Resources, Hawassa University, Postbox 128, Shashemene, Ethiopia; 3 Norwegian Institute of Bioeconomy Research (NIBIO), Postbox 115, NO-1431 Ås, Norway; 4 Antec Biogas As, Olaf Helsets Vei 5, 0694 Oslo, Norway; 5 Faculty of Chemistry, Biotechnology and Food Science (KBM), Norwegian University of Life Sciences (NMBU), Postbox 5003, 1432 Ås, Norway; 6 Department of Process, Energy and Environmental Technology, University of South-Eastern Norway (USN), Campus Porsgrunn, Kjølnes Ring 56, 3918 Porsgrunn, Norway

**Keywords:** Biogas, Methanogens, Biomethane, Full-scale biofilm reactor, Lignocellulosic biomass

## Abstract

Biofilm is a syntrophic community of microorganisms enveloped by extracellular polymeric substances and displays remarkable adaptability to dynamic environments. Implementing biofilm in anaerobic digestion has been widely investigated and applied as it promotes microbial retention time and enhances the efficiency. Previous studies on anaerobic biofilm primarily focused on application in wastewater treatment, while its role has been significantly extended to accelerate the degradation of lignocellulosic biomass, improve gas–liquid mass transfer for biogas upgrading, or enhance resistance to inhibitors or toxic pollutants. This work comprehensively reviewed the current applications of biofilm in anaerobic digestion and focused on impacting factors, optimization strategies, reactor set-up, and microbial communities. Moreover, a full-scale biofilm reactor case from Norway is also reported. This review provides a state of-the- art insight on the role of biofilm in anaerobic digestion.

**Graphical Abstract:**

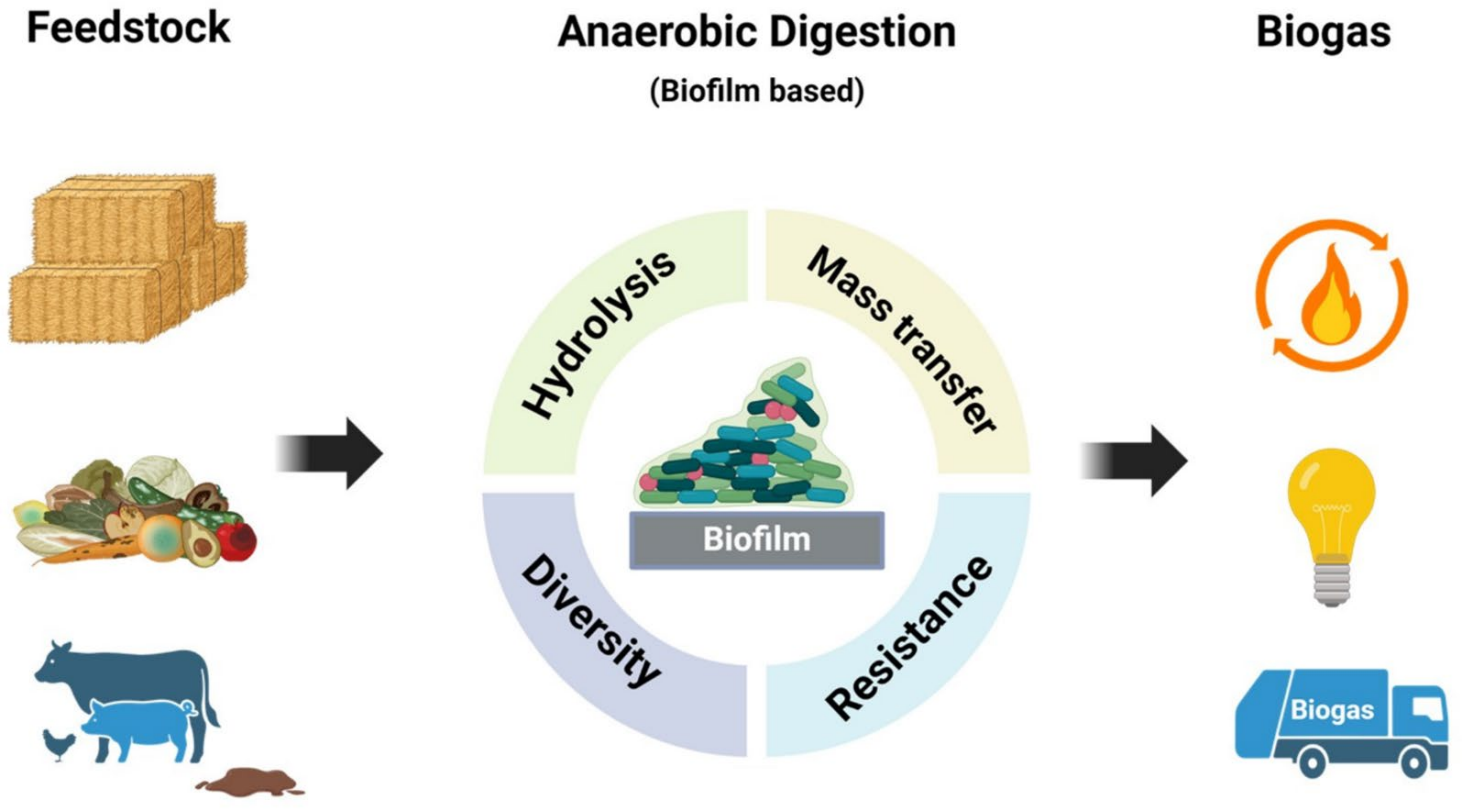

## Introduction

Anaerobic digestion (AD) is a biological process that breaks down organic wastes in the absence of oxygen (O_2_) to generate biogas (containing methane (CH_4_), carbon dioxide (CO_2_) and other gases in smaller concentration) and a nutrient-rich residue, so-called digestate, which can be used as fertilizer [1]. It is applied worldwide to valorise organic waste, e.g., sewage sludge, food waste, and animal slurry while minimizing its negative environmental impact [2]. Despite these benefits, the development of AD and biogas sector still face challenges due to the low conversion rate, poor process stability, low economic value for final product [3], while application of biofilm is a promising option to optimize AD.

The formation of biofilm is a multistage process where microorganisms adhere to and grow on the support or carrier material by producing extracellular polymeric substances (EPS) [4]. This consortium comprises of microorganisms such as archaea and bacteria working synergistically [3]. The consortium develops metabolic and functional diversity, boosting its survival under dynamic conditions compared to its planktonic counterparts [5]. Application of biofilm to AD offers several advantages. First, the significant advantage is the extended biomass retention time in the system, helping slow growing anaerobic microorganisms which can take days to weeks [6]. The extended retention of microbial biomass serves as an important basis for achieving effective and robust waste degradation [7]. Second, biofilm also improves the mass transfer of gas–liquid, nutrients through large surface area, yielding high local microbial activity [8]. Recent studies also demonstrated promising results in enhancing the methanogenesis process through developing biofilm-based processes, particularly in utilizing CO_2_ and H_2_ [9, 10]. In this context, biofilm reactors prove to be solution for enhancing the efficiency of H_2_ gas mass transfer compared to non-biofilm AD [11]. In addition to these benefits, biofilm-based reactor could establish a dense population of selective microorganisms that enhances the resistance to inhibitors, for instance excess ammonia or accumulation of volatile fatty acids (VFAs). These improvements are achieved with lower energy input, in doing so increase biomethanation processes [13]. Some EPS possess ion exchange membrane that is able to bind pollutants and metals, which is valuable for waste treatment [14]. Nevertheless, its application in AD still faces challenges, such as biofilm formation, instability, and biomass washout. The mechanisms underlying these obstacles influenced by factors such as variation in hydrodynamics, hydraulic retention time (HRT), solid retention time (SRT), and reactor configuration and design [15]. For example, the variation in the hydrodynamics of waste could cause a variation in shear force affecting biofilm attachment and stability, leading to limitations in mass transfer and nutrient availability [16]. Therefore, ensuring sustained biofilm integrity and preventing detachment during dynamic operational conditions are fundamental aspects to address for optimizing biofilm-based AD processes [4, 7]. There are currently increasing focus on mixed-species biofilms rather than strict anaerobic biofilm for AD. Shahab et al. [17] constructed a multispecies biofilm in which aerobic fungus (*Trichoderma reesei*) formed a biofilm that secreted cellulolytic enzymes and facultative anaerobic bacteria (lactic acid bacteria) in the liquid medium led to a better yield of short-chain fatty acids (SCFA) from lignocellulosic material. Therefore, these new knowledge and progress should also be reviewed and updated.

In general, application of biofilm in AD has experienced significant growth over the past decade. Most published reviews, however, focused on only wastewater treatment, with very limited attention given to other feedstocks, purposes, or applications. There is also a lack of insight into practical performance as most of the publications primarily report laboratory-based results. In this review, we aim to provide an overview of the latest advancements in the utilization of biofilms in AD. Furthermore, we include an industrial scale biofilm reactor to give insights into the practical application.

## Biofilm formation and mechanism

### Biofilm formation

**Fig. 1:**
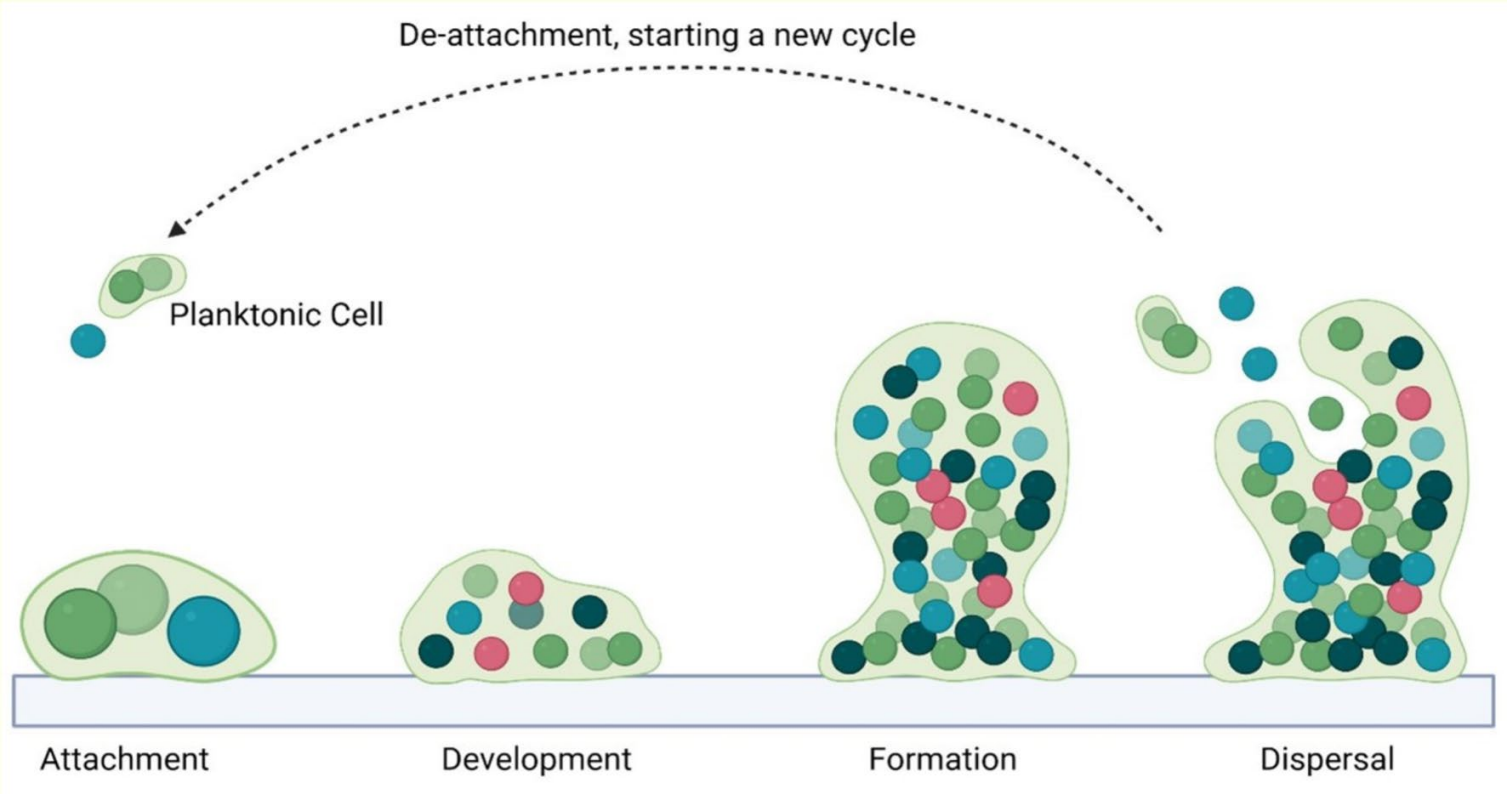
Stages in biofilm formation. (The figure is adapted according to [21])

Biofilm formation begins with the organized attachment of microbes to accessible surfaces, carrier materials, and sludge flocs. This attachment is facilitated by the production of EPS, which contains components such as polysaccharides, proteins, lipids, and nucleic acids that aid in microbial adhesion [18]. It is a survival mechanism for microorganisms under hostile environmental conditions and function as an adaptive strategy that enables them to persist within a given ecosystem [19]. The process is driven by the production of quorum-sensing molecules (auto-inducers) that initiate microbial communication for biofilms formation [20]. The cycle involves microbial attachment to accessible surfaces, microcolony development, biofilm formation, and finally dispersal (Fig. 1) [21]. The colonization and biofilm formation depends on factors such as wettability, surface area, porosity, texture, and electrical conductivity of the accessible surfaces or carrier materials [18]. Formation of biofilm increase the microbial population and diversity, leading to higher metabolic activity and syntrophic relationship within the biofilm. The matrix also offers protection against adverse environmental conditions by creating a physical barrier and facilitating genetic exchange [19, 22]. Moreover, the higher moisture content around it creates a nutrient gradient that enables the movement of nutrients towards the biofilm through the diffusion and concentration process [23]. This inward movement of nutrients ensures efficient supply to the thriving microbial community within the biofilm [22]. During the onset of adverse environmental conditions, such as peak population level and nutrient depletions, the cells within the biofilm respond by producing hydrolytic enzymes to breakdown the matrix, leading to detachment and transition to a planktonic form [22, 24]. For instance, the bacterium *Pseudomonas aeruginosa* produces a well-known biofilm dispersal inducer molecule (quorum quenching), cis-2-decenoic acid, in response to resource limitation and environmental stress, influencing cells from the matrix [25]. Subsequently, the detached microorganisms re-enter to the environment, begin colonizing new surfaces, create new biofilms, and maintain the cycle [19].

### Biofilm application in AD

Biofilms have proven to be beneficial in AD processes, which facilitate the degradation of lignocellulosic biomass, extend biomass retention time, reduce the lag phase, enhance microbial resistance to inhibitors, and improve the mass transfer [7, 26]. Table 1 lists the overview of biofilm application in AD and summarises the purpose and mechanisms.

**Table 1:** Overview of biofilm applications in AD, the purpose, and potential mechanisms

No	Application of biofilm	Mechanism	Reference(s)
1	Enhanced hydrolysis of lignocellulosic materials	Microbial functional diversity and synergy with complementary capability, concentrations of enzymes around biofilm matrix (such as cellulases and hemicellulases), concentrating nutrients, increased activity of genes responsible for producing carbohydrate active enzymes	[26, 27]
2	Improved mass transfer	Large surface area having three-dimensional structure, improved waste diffusion and osmosis through their higher porous structure	[8, 13]
3	Enhanced process stability and efficiency	Prolonged microbial biomass retention time, resilience to inhibitors, higher microbial density	[7, 29]
4	Boosted resistance to inhibitors	Physical barrier against inhibitors, creating nutrient gradients, population heterogeneity, efflux pumping of toxin, and detoxification, slower growth	[30, 31]
5	Steady and efficient gas fermentation	Ideal environment for methanogens, enhanced syntropy and diversity, long-term cell activity resistance to toxic reactants, improved mass transfer, and mitigating microbial washout	[22, 28]

#### Enhance the hydrolysis rate

Lignocellulosic biomass generated from agriculture sector, such as straw or grass, represents one of the most abundant bioresources for biogas. The main challenge of using them lies in their recalcitrant nature, making difficulties for hydrolysis and subsequent biological conversion [26]. To address this, the utilization of multispecies biofilm has demonstrated promising results to improve the hydrolysis process [32]. The complementarity in metabolic and functional diversity within multispecies biofilms enable the successful degradation of lignocellulosic biomass. To illustrate, combining biofilm-forming bacteria such as *Clostridium thermocellum* with cellulose-degrading fungi such as *Trichoderma reesei* has the potential to enhance enzyme production and improve cellulose and xylene degradation and under anaerobic and microaerobic conditions [22, 33]. Xiros et al. [33] showed enhanced cellulose hydrolysis by co-cultivation of cellulose-degrading fungal biofilm with a consortium of rumen microbes in a multispecies biofilm reactor. This synergy resulted in a 39% higher SCFA production compared to the production achieved by the rumen microbiome alone. Similarly, Shi et al. [34] reported an increase in the degree of acidification by 51.6% using an integrated floating film and activated sludge system equipped with zero-valent iron composite carriers in the anaerobic treatment of dairy wastewater, which is a 9.5% increase compared to reactors without biofilm carriers. Moreover, the application of biofilms to AD could improve the hydrolysis of waste with a high chemical oxygen demand (COD) and concentrated waste such as municipal waste. Shi et al. [35] demonstrated the effectiveness of anaerobic biofilm combined with thermal alkali pretreatment to improve the hydrolysis of sludge with high-solid and low organic content, extending beyond lignocellulosic biomass. In this case, biofilm increased the population of fermentative microbes which subsequently enhances both hydrolysis and acidogenesis processes.

#### Increase resistance to inhibitors

In addition to CO_2_ and CH_4_, AD can result in the generation of various intermediates or impurities, including VFAs, alcohols, ammonia (NH_3_), hydrogen sulphide (H_2_S), etc. (Table 2). The buildup of these compounds within the reactor affects process performance and stability. For instance, the accumulation of VFAs, mainly acetic and propionic acids, can cause a decrease in pH and operational instability [36]. Similarly, the AD of high sulphide/sulphate containing wastes, such as wastes from slaughterhouses and tanneries, could result in the production of H_2_S along with other biogas constituents [37]. It was also reported that a H_2_S concentration above 300 ppm could completely inhibit the activity of methanogens and promote sulphate-reducing bacteria (SRB) to outcompete methanogens [38, 39].

**Table 2:** Factors inhibiting AD and microbial response mechanisms

Inhibitor	Impact on microbial activity	Microbial response	Reference(s)
Salinity	Inhibition of cell growth, reduced cell metabolism, Osmotic imbalance, reduced biofilm formation	Production of EPS acts as physical barrier, salt tolerance, adaptation, compatible solute production	[45, 46]
Antibiotics	Cell wall disruption, interference with protein synthesis, disrupting metabolic activity etc	EPS acts as physical barriers, modification of lipopolysaccharide, slow growth, and impairing antibiotics uptake, physiological and genetic diversity, physiological limitation	[19]
pH	Inhibition of microbial activity	EPS serves as buffer, physical barrier, inhibition of toxic formation by EPS	[19, 36, 47]
Temperature	Affect metabolic activity, destroy biomolecules	EPS serves as physical barrier or protective clothing	[19, 36, 47]
H_2_S	Inhibits cell growth and enzymatic activity, Enables SRB to compete with the consortia member, and toxicity	Tolerance, enzymatic response, genetic response, adaptation of metabolically outcompeting of SRB, quorum sensing	[37, 48]
NH_3_	Inhibition of methanogenesis, reduction of pH due to accumulation of acetate, and propionate, changing methanogenesis pathway	Metabolic adaptation to ammonia stress, ammonia efflux, metabolic shift	[49–51]

Establishing of biofilm offers advantages in coping with these inhibitors, including creating physical barriers through matrix development, limitation of physiology, gene diversification, modification of lipopolysaccharide, hinder antibiotic uptake, and detoxification [19, 40]. These features significantly enhance resistance against inhibitors, preventing biofilm detachment, and ensuring the structural integrity of AD systems. This protective function is attributed to the matrix formed by the EPS, along with the spatial and chemical heterogeneity it introduces [41]. Moreover, biofilms not only protect microorganisms from hostile environments, but also introduce synergetic relationships and help horizontal gene transfer [42]. This resilience is key to the sustained functionality of AD systems. Research suggests that microorganisms embedded within a biofilm can evolve up to three orders of magnitude more tolerant to antibiotics compared to free-living forms [31]. Dykstra and Pavlostathis [43] reported the resilience of biofilm in a bioelectrochemical system against H_2_S inhibition and observed that CH_4_ production increased with the H_2_S concentration rose from 0 to 2% (*v*/*v*). It should be noted that this concentration of H_2_S is significantly higher than the concentration typically found in raw biogas. Sella et al. [44] used anaerobic structured bed biofilm reactors for treating wastewater containing sulfamethoxazole (SMX) and demonstrated up to a 90% SMX removal rate using acclimatized inoculum from poultry slaughterhouse sludge. This finding denotes the requirement to select an appropriate inoculum and acclimatize microbes for better waste degradation.

#### Enhanced nutrient transfer

Enhancing waste degradation and microbial activity in AD can be influenced by optimizing nutrient and solute transport efficiency. The mass transfer efficiency is affected by factors, such as mixing limitations, flow characteristics, viscosity, and the solid content of the waste stream [52]. These factors can create inadequate contact between waste and microorganisms, resulting in decreased biodegradation, accumulation of inhibitory compounds, limitation of nutrients, fluctuations in pH, and a prolonged residence time of waste [52]. Anaerobic biofilm reactors present a promising solution to enhance the mass transfer issue [11]. The biofilm forms a three-dimensional structure characterized by nutrient gradients, biofilm streamers, increased inward diffusion of wastes, and improved mass transfer. Taherzadeh et al. [8] highlighted the importance of the periodic oscillatory movement of biofilm streamers (filaments) in boosting nutrient mass transfer rates. Specifically, vibrating biofilm streamers resulted in an increase of 11% in the mass transfer rate compared to the stationary state. Thick, low-density biofilms characterized by porous and rough properties have demonstrated effectiveness in enhancing mass transfer coefficients. In relation to this, Beyenal and Tanyolaç [53] reported increase in thickness and decrease in density of biofilm significantly raised the mass transfer coefficient to 7 × 10^–4^ ms^−1^, specifically when the biofilm thickness reaches around 100 µm. Similarly, Brito and Melo [54] studied the influence of anaerobic biofilm formation on the enhancement of the mass transfer coefficient in a range of fluid velocities (between 1.5 and 13.2 m/h) in the context of wastewater treatment. Their study revealed a steady-state value in the final period of biofilm formation in the range of 2–4 × 10^–3^ m/h and resulting in an average 20% boost in the internal mass transport coefficient.

#### Biological biogas upgrading (biomethanation)

Recent years, there has been a growing trend worldwide to employ methanogens to convert H_2_ from renewable sources and CO_2_ to generate biomethane, namely biomethanation [10]. This approach offers the prospect of energy storage at a low investment cost, so-called power to gas. However, the effectiveness is impeded in regular process due to the low solubility of H_2_ and poor mass transfer. To mitigate the issue, biofilm-based ADs, typically dominated by hydrogenotrophic methanogens, is intensively investigated [55]. For instance, Miehle et al. [56] reported achieving a biomethane concentration of up to 99% using a membrane biofilm reactor during ex situ hydrogenotrophic methanation with an H_2_ and CO_2_ with a stoichiometric ratio of 4.12:1. Pratofiorito et al. [12] examined the influence of archaeal biofilm formation on the biomethanation of biogenic CO_2_ using a custom-made membrane biofilm reactor and led to an increased CH_4_ content up to 97% (v/v). Savvas et al. [10] used a biofilm plug flow reactor for biomethanation inoculated with mixed consortium and achieved over 99% biomethane. Likewise, Thapa et al. [57] reported successful biomethanation of up to 93.5% CH_4_ utilizing an H_2_:CO_2_ stoichiometric ratio of 6:1, while Tauber et al. [58] achieved up to 98.0% CH_4_ using a TBR. In another experiment, Maegaard et al. [55] studied the role of hydrogenotrophic methanogenic biofilm formation on a carrier material in increasing the conversion of H_2_ and CO_2_. The results showed that biofilms grown on carrier material produced a CH_4_ content of up to 95%. Jensen et al. [13] also found that biofilm biomethane production increased by a factor of 5.4 to 12.5 and CH_4_ concentration reached above 92% when operating at a shorter hydraulic retention time (HRT) of 18 h compared to a longer HRT of 20 days when biofilm had been established. Biofilm could significantly enhance the mass transfer from gas to liquid phase, which influence not only H_2_/CO_2_ to CH_4_ but also CO from syngas generated from pyrolysis. Shen et al. [59] reported a significant enhancement in the CO gas–liquid mass transfer coefficient of 1096.2/h using hollow fibre membrane biofilm reactors, which was found to be higher than the mass transfer coefficient of 72.8/h in the CSTR reported by Younesi et al. [60]. Asimakopoulos et al. [61] demonstrated efficient syngas fermentation using a biofilm developed in a trickle bed reactor (TBR) and achieved a conversion rate of 93% for H_2_ and 90% for carbon monoxide (CO), respectively. The improvements in gas–liquid mass transfer, extended microbial retention and stability, and suitability of biofilm environments for hydrogenotrophic methanogens could have contributed to the improved efficiency of biofilm-based AD systems during biomethanation processes.

## Factors affecting biofilm-based AD process

The formation of biofilm can be affected by several parameters or factors, including temperature, pH, competition and co-existence, shear force, nutrient availability, carrier material and HRT [97]. These parameters or factors could either prevent, inhibit, or slowdown the formation of biofilms or accelerate the detachment of biofilms formed. Optimizing these factors has significant impact in fostering the development of biofilms that are essential to efficient AD.

### Temperature

Temperature is often considered an important factor affecting microbial community, the formation of biofilms, and AD process [62, 63, 97]. The AD process is often classified as thermophilic (50–60 °C), mesophilic (30–40 °C), and psychrophilic (< 30 °C), while most of AD are operated at mesophilic or thermophilic temperatures [64]. It influences the growth and metabolism of microbes involved in the biofilm, with the activity of these consortium members that determining the efficiency and stability of AD [63]. Moreover, temperature affects the rheology of the EPS produced, decreasing its viscosity, and affecting its gluing property during matrix formation [65]. Levén et al. [66] reported that the operating temperature of a digester leads to a remarkable variation in archaeal and bacterial community composition and diversity, where mesophilic temperatures found to host relatively higher diversity of microbial community compared to thermophilic temperatures. This variation in community composition and diversity affects performance of AD. Patil et al. [67] reported that raising the temperature from 22 to 35 °C accelerated the biofilm formation by three-fold and reduced the lag time by 71% (from 12 to 3.5 days). Furthermore, Zhao et al. [68] examined the impact of decreasing processing temperature from mesophilic (30 °C) to psychrophilic (3 °C) on the activity of microbial community growing on carbon fibre and sludge. This decreased temperature led to an accumulation of VFA and a decrease in biogas production from 3.71 to 0.04 L/kg COD/m^3^/days. This suggests that instability in the operating temperature could affect the microbial community and the efficiency of AD, necessitating for continuous monitoring and regulation of temperature.

### pH

The microbial activity is sensitive to pH, which can manifest its influence by directly affecting the microbes engaged in the process or by altering the chemical equilibria of various by-products, including VFA, H_2_S, NH_3_ and others. The optimal pH range for AD is in the range of 6.5–8.0 [69, 70]. Deviations from this range can significantly affect microbial enzymatic activity, cell membrane integrity, protein denaturation, nutrient solubility and availability, growth inhibition, and even lead to cell death [71]. Several factors such as high OLR, imbalanced buffering capacities, and specific feedstock compositions, can contribute to deviation in pH [72]. This deviation in pH from optimal range have the potential to disrupt microbial cellular functioning and molecular processes [73]. This decrease in microbial activity and metabolism could potentially alter biofilm related parameters.

Multiple studies showed that pH deviations from optimal ranges could affect the metabolic functioning. For example, Zhang et al. [74] explored the impact of pH variation on the hydrolysis and acidogenesis stages of two-phase anaerobic biodegradation of kitchen waste in batch and semi-continuous setups. The results showed significant improvement in the hydrolysis and acidogenesis stages at pH = 7 (with 86% total organic carbon and 82% COD solubilization) compared to pH = 5, 9, and 11. Similarly, Gutierrez et al. [75] reported the effect of prolonged pH elevation (at levels of 8.6 and 9.0) on the activity of SRB and methanogens within anaerobic sewer biofilm. The results indicated a significant reduction in SRB activity by 30% and 50% at pH = 8.6 and 9.0, respectively, compared to the 6.4 ± 0.4 mg S/L hour in the control. However, the methanogenic activity was not significant, with a rate of less than 0.7 mg CH_4_ COD/L per hour at both pH levels, which is considerably lower compared to the 21.4 ± 0.3 mg CH_4_ COD/L per hour production observed in the control.

Moreover, Nostro et al. [76] demonstrated the effect of increasing pH from 7.2 to 8.5 over different incubation periods (3–24 h) by *Staphylococcus aureus* and noted a decrease in the density of the biofilm. pH variation also affects the EPS production. Likewise, Solmaz et al. [77] reported the impact of varying pH levels (6.5, 7.0, 7.5, 8.0, and 9.0) on the ability of *Bacillus pseudomcoides* to produce EPS and found that pH = 7 was optimal. The microbial community involved in AD varies in their pH requirements. Understanding and optimizing the pH needs of the respective groups is crucial for the digestion process to occur efficiently.

### Competition and co-existence

As a biological process, AD is carried out by hydrolysers, acidogens, acetogens, and methanogenic archaea, working synergistically to facilitate the conversion [78]. However, its efficiency is also affected by competition between microbes existed within AD system. For example, SRB engage in competition with acetogens and methanogens for available nutrients. These microbial groups compete for available resources during the degradation of sulphate-rich feedstocks, which can inhibit the growth of methanogens [79]. According to Sela-Adler et al. [80], co-incubation of SRB and methanogens with acetate and lactate in a range of sulphate concentration led to a two-order of magnitude reduction in methanogenesis rate (µmol L^−1^ day^−1^). In this situation, SRB uses sulphate as a terminal electron acceptor and outcompetes methanogens for available nutrients. Dar et al. [79] observed the dominance of SRB in feedstock containing a lactate-to-sulphate ratio of 0.35 mol/mol, which promoted the growth of SRB growth, constituting over 80% of the microbial population. This competition directly influences on biofilm formation and the microbial community. Despite the competition between SRB and methanogens, Shi et al. [81] found a mutual association between the microorganisms after an extended co-incubation period. The SRB produced Methyl-coenzyme M as a product of their metabolism, which serves as an essential compound for methanogens. In addition, competition also arises between methanogens and acetogens. The outcome of such competitions depends on the concentration of nutrients, with methanogens tend to gain an advantage under limited nutrient conditions. Florencio et al. [82] reported that methylotrophic methanogens dominate acetogens under condition of low methanol or inorganic carbon, while acetogens dominate at high methanol concentration (> 1000 mgCOD/L).

Further studies on competition and coexistence in biofilms within AD systems stress the importance of maintaining balanced feedstock compositions. Raskin et al. [83] examined the competition and coexistence between SRB and methanogens within a biofilm following the addition of sulphate and glucose containing substrate. They reported that in the presence of glucose alone, methanogens comprised up to 25% of the population, while SRB made up to 15%. However, with the addition of sulphate to the glucose, the SRB population increased to 30–40%, whereas the methanogen population decreased to 8%. Likewise, Yoda et al. [84] examined the long-term competition between SRB and methanogens for acetate in a sulphate-containing medium within an anaerobic biofilm. They found that when the influent sulphate concentration was 145 mg L^−1^, methanogens utilized most of the acetate, leading to a CH_4_ production of 1397 mL day^−1^. However, as the influent sulphate concentration increased to 232 and 400 mg L^−1^, CH_4_ production decreased to 1021 and 508 mL day^−1^, respectively. These findings suggest that SRB have a broader substrate preference in sulphate-rich substrates compared to methanogens. Therefore, it is crucial to maintain a balanced feedstock composition, supplement nutrients appropriately, and create optimal operational conditions to avoid competition and promote co-existence.

The presence and availability of H_2_ also influence the competition between SRB and methanogens. In most anaerobic environment, H_2_ serves as an intermediate for SRB, hydrogenotrophic methanogens, and homoacetogens [79]. Under standard conditions, sulphate reduction and methanogenesis dominate over homoacetogenesis. When H_2_ is limited and sulphate is abundant, SBR outcompete hydrogenotrophic methanogens and become the primary H_2_ consumers [164]. In scenarios where H_2_/CO_2_ are the only substrates, such as ex-situ biomethanation, SRB rely on homoacetogens [165]. Consequently, the growth of homoacetogens determines the competition between SBR and hydrogenotrophic methanogens. Weijma et al. [164] investigated the competition for H_2_ between SRB, methanogens and homoacetogens in a continuous gas lift-reactor. The researchers reported that when the H_2_ loading was higher than sulphate (H_2_/SO_4_^2–^ at a ratio of 12 mol/mol), there was no competition between the microorganisms for H_2_. However, when the ratio of H_2_/SO_4_^2–^ loading was decreased to 2.5 mol/mol, the CH_4_ production reduced by 80% on day 5 and 98% on day 20, indicating SRB outcompeted methanogens for H_2_.

### Shear stress

The intensity of shear stress under different hydrodynamic conditions has impact on attachment of microbes to carrier material and proliferate [85]. In contrast to low shear intensity, higher shear stress can lead to the growth of thinner, compact, and stronger biofilms that take longer time to mature and display a reduction in microbial diversity [86]. This property may result in a decrease in the porosity of the biofilm, which in turn could impact mass transport and potentially affecting efficiency of waste treatment process. In this regard, Rochex et al. [87] examined the effect of varying shear stress levels (between 0.06 and 0.27 Pa) on biofilm property and found that high shear stress resulted in a decreased diversity and slower maturation of biofilm. Pechaud et al. [88] also found that an increase in shear intensity from 0.5 to 9.0 Pa led to a decrease in the biofilm thickness from 4200 to 250 µm, respectively, and an increase in the density of the biofilm.

The impact of shear stress on biofilm development and detachment also affected by the nature and type of surface material used. Lackner et al. [89] demonstrated the application of polypropylene membranes and polyethylene surface modification with polyethyleneglycol chains that have amino groups. These modifications were shown to enhance biofilm formation and increase the shear resistance of nitrifying bacteria. The development, thickness, and proliferation of biofilm reduce as shear forces go beyond certain thresholds [90]. Increased shear stress has also been shown to interfere with cell-to-cell communication, which is essential for biofilm development and the attachment of biofilm components [85]. Therefore, it is crucial to optimize the hydrodynamics of waste for relevant process operations.

### Nutrient

Nutrients supplement is essential for biofilm development in AD, as they are vital for enzymatic activities, cell structure and function, and energy production in microorganisms [91]. The microorganisms involved in AD require a variety of macro- and micronutrients for their growth and metabolism (Table 3) [92]. The macronutrients essential for supporting microbial growth are needed in higher amounts, whereas the micronutrients are needed in lower amounts [93]. In addition to the nutrients, vitamins such as Vitamin B1, B2, B3, B6, B7, and B12 are necessary in AD, as they are involved in the methanogenesis process, with cobalt serving as a methyl acceptor cofactor [94]. These nutrient and vitamins can be supplied through the substrate fed into the reactor or during an inoculum change. In addition, it is also possible to acquire low-cost nutrients, vitamins, and trace metals from resources, such as cow manures for AD [95]. Izadi et al. [94] found that the combined addition of micronutrients and vitamins led to an increase of up to 30% in CH_4_ production compared to the only addition of vitamins and the control group.

**Table 3:** Macro- and micronutrient composition for supporting microbial growth in AD [93]

Nutrients		Function
Macronutrient	Nitrogen, Phosphorus, Sulphur	Formation of cellular biomolecules (DNA, RNA, FAD + , NADP + , ATP, protein, amino acids etc.)
Micronutrients	Iron, Cobalt, Zinc, Selenium, Tungsten, Magnesium, Chromium, Nickel, Molybdenum	Stimulation of cellular metabolism, growth factor for acetogens, synthesis of cofactor III, production of carbonic anhydrase and others

Wang et al. [96] evaluated the morphology of biofilm under nutrients rich condition (also consisting of nitrogen and phosphorus), and or nutrients limited conditions. The biofilm exhibited a heterogeneous structure under nutrients rich condition with large clusters of microbes with a diameter of around 5 µm. Under nutrients limited condition, there were fewer scattered microbes (nitrogen limited) or a thinner and denser biofilm (phosphorus limited). In another study, Mei et al. [97] observed a decrease in the dry weight of biofilm formed by hydrogen-producing bacteria when the glucose concentration exceeded a certain threshold, increasing from 5 to 45 g/L.

In addition, nutrient availability is affected by OLR and the feedstock. An imbalanced nutrient can cause process instability and accumulation of inhibitory compounds. Therefore, ensuring an adequate amount of organic substrate entering the system is crucial in providing microbes with sufficient access to nutrients. For instance, Cresson et al. [91] observed that increasing the OLR from 0.5 to 6 gCODL^−1^ day^−1^ led to the limitation of essential micronutrients such as cobalt and nickel, resulting in the accumulation of volatile fatty acids (VFA) and other inhibitory metabolites. This negatively affected reactor performance and methanogenic biofilm activity. However, the supplementation of micronutrients restored optimal methanogenic activity and improved COD removal.

### Carrier materials

The selection of carrier materials is important in biofilm formation. These materials should exhibit specific characteristics such as good wettability, feasible shape, adequate porosity, optimal size, high surface area-to-volume ratio, and a rough texture, which influence microbial attachment and the maturation of biofilms [98–100]. Zhou et al. [101] reported that hydrophilic polymeric carrier materials tend to favour/promote sustained biofilm formation over time. Setiyawan et al. [102] examined the impact of wettability of carrier materials on biofilm dynamics and reported that hydrophilic materials, such as polyethylene terephthalate, resulted in increased total attached solid, accelerated biofilm formation, and thicker biofilm. The physical property of carrier material also exerts a significant influence on biofilm formation. Dias et al. [103] examined the influence of carrier material characteristics on biofilm formation and reported that spherical-shaped carrier materials with larger pore size required shorter duration to reach stable biofilm formation (from 15 to 17 days) compared to the cylindrically shaped biofilm carriers with smaller pore size (from 23 to 24 days). This finding suggests the influence of geometry and porosity of carrier materials play a role in shaping biofilm dynamics. Moreover, Ahmad et al. [99] studied the influence of surface area, size, and roughness of carrier materials on biofilm development. Their findings indicated that carrier materials with a larger surface area (1200 m^2^/m^3^) and higher surface roughness facilitated better biofilm adhesion, resulting in a biofilm thickness of 2250 µm compared with smaller surface area (500 m^2^/m^3^) and less roughness (biofilm thickness of 488 µm). Smaller-sized carriers resulted in a loose biofilm, whereas carriers with a size of 15 mm showed good texture and biofilm accumulation. In conclusion, the selection of carrier material is fundamental to promote biofilm formation in AD.

### Hydraulic retention time (HRT)

Sufficient HRT facilitates extensive interactions between microorganisms and waste, promoting microbial growth, and supporting biofilm formation. Previous research emphasized that HRT could impact waste treatment, shape microbial community, and improve biofilm development [88, 104]. Pechaud et al. [88] reported that lower HRT (3 h) enhances biofilm and streamer formation, and thickness compared to higher HRT (20 h). Research has emphasized the significance of shorter HRT for the development of biofilms. Peces et al. [105] reported a significant decrease in microbial diversity and the dominance of a few species and increase in biofilm formation when the HRT was reduced from 15 to 2 days. This reduction in HRT also resulted in a decrease in methane yield and an increase in VFA accumulation. HRT also has an impact on biofilm development. Mei et al. [97] examined the influence of HRT, nutrient concentration, and inoculum source on biofilm formation and observed significant increment of dry weight of biofilm from 4.1 to 21.7 g/m^2^ when HRT extends from 3.5 to 8 days. Shen and Zhu [106] reported that the highest volumetric CH_4_ production rate was 2.95 L/day at an HRT of 8.57 days and decreased beyond this duration. Similarly, Song et al. [107] reported the highest COD removal efficiency (over 70%) at a longer HRT of more than 20 h, compared to a lower HRT, which achieved a 50% COD removal rate. These findings suggested that HRT enhanced the efficiency of biofilm. Zhang et al. [108] investigated the effects of varying sludge retention time (10, 15, 20, and 30 days) and reported that a longer SRT promoted biodiversity, increased stability, and resulted in higher CH_4_ content. The CH_4_ content increased from 251 ± 11 to 302 ± 25 mL/g VSS as the retention time increased from 10 to 30 days.

## Anaerobic biofilm reactor

### Different kinds of biofilm reactors

Anaerobic biofilm-based reactors utilize biofilm growing on the internal surface of reactors, carrier materials, or sludge particles to enhance the performance. The higher density of resilient and robust microbes embedded within the biofilm serves as a catalyst in the reactors. Moreover, these reactor types are characterized by the ability of working with higher organic OLR, achieving higher waste removal rate, and demonstrating resilience to shear forces. This enhances the speed and efficiency of waste degradation by the system [109]. These reactors can be of various types, designs, and configurations depending on their purpose (shown in Table 4). Some of these reactor types include up-flow anaerobic sludge blanket (UASB) reactors, plug-flow reactors, CSTR, inverse turbulent bed reactors, packed bed reactors, fluidized bed reactors, and dynamic membrane reactors [24, 91, 110]. Each of these reactor types has distinct features and advantages, and the selection of reactor type can influence the operation of the AD process.

**Table 4:** Characteristics of anaerobic biofilm reactors and carrier materials

Reactor type	Feature	Carrier material	Reference(s)
UASB reactor	High-rate AD system, possess granular sludge flocs, easy separation of sludge, best work for low and high strength wastes, high COD removal, low sludge production, heavy metal treatment, retain high biomass concentration, perform at lower HRT	Sludge blanket as biofilm carrier	[111, 112]
Anaerobic up-flow fluidized bed reactors	Higher COD removal and better CH_4_ yield, higher nitrate removal, better mass transfer, higher attached biomass, treatment of dairy waste	Pumic stone, Saponite, sludge particles, Gravel, charcoal, Granular activated carbon, low-density PVC,	[109, 113]
Anaerobic down-flow fluidized bed reactors	Efficient COD removal and higher CH_4_ yield, treatment of dairy waste, less energy requirement	Polystyrenes, anthracite, Granular Silica, high density plastic beads, and aluminium, sand, polyethylene, polystyrene ball packing material	[109]
Anaerobic fluidized bed biofilm reactor	High COD removal rate, higher biomass concentration and lower microbial washout, higher CH_4_ yield, improve contact time between microbes and waste to be treated	Kaldness-K1, Sepiolite, perlite	[114, 177]
Anaerobic migrating blanket reactor (AnMBR)	Higher specific methanogenic activity, highly efficient for low organic loadings, reduced sludge production, low energy requirement, efficient COD removal (98%)	Sludge particles as carrier material	[115, 116]
Anaerobic membrane bioreactor	Reduced sludge, higher biogas production, higher COD removal, synthetic and municipal waste treatment, high sludge retention time	Membrane module	[117]
Anaerobic fixed film baffled reactor	High biomass accumulation, enhanced solid separation, shorter HRT, reduced sludge, efficient COD removal, efficient for low to highly hazardous pollutants	High density polyethylene carriers, reactors′ surface/compartment, sludge blanket	[118, 119]
Anaerobic rotating biological contactor (AnRBC)	Low energy requirement, best for high strength wastes, high organics, nitrogen and ammonia removal,	Submerged rotating discs	[120, 176]
Anaerobic sequencing batch reactor (AnSBR)	Extended microbial retention, high rate of waste conversion, high biogas production, removal of high strength waste, effective BOD, COD and TSS removal	Natural zeolite, granular activated carbon, low-density polyethylene, textile-based carrier, ceramics, fiberglass	[121, 122]
Anaerobic moving bed biofilm reactor	Higher soluble COD and BOD removal, high biomass/sludge retention	PVA gel beads, AC920 plastic media	[123, 124]
Anaerobic trickling filter reactor	Higher pollutant removal, higher methane yield, improved gas-to-liquid mass transfer, suitable for biogas upgrading	Rocks, polymeric carrier materials	[61, 125]
Anaerobic expanded granular sludge bed reactor	Stable waste treatment efficiency, higher pollutant removal rate, high biomass accumulation, high treatment efficiency	Sludge particles or anaerobic flocs or clumps	[126]
Anaerobic dynamic membrane bioreactor (AnDMBR)	Higher COD (99%) and organic removal efficiency, low sludge production, cheap membrane module, high biomass accumulation	Dynamic membrane module, polypropylene monofilament woven fabric, activated carbon, flocculant	[127, 128]
Biofilm supported CSTR	Higher COD removal, higher (25% more) methane yield	Low-density nylon meshes, fibrous bed support, polypropylene	[129]
Biofilm plug-flow reactor (BPFR)	Higher methanogenesis with efficient CO_2_ conversion	High-desnity polyethlene (e.g., Kaldnes) k1	[10]

The formation of biofilm in anaerobic biofilm reactors occurs through the use of biofilm carriers, facilitating microbial attachment to the internal surface of the reactors, and fostering the adhesion of microorganisms on anaerobic sludge aggregates [7, 24].

### Biofilm formation

#### Formation on carrier materials

Carrier materials play an essential role in anaerobic biofilm reactors. A variety of carrier materials, including both organic and inorganic types, have been employed to foster the formation of biofilm (Table 5). (The stages of biofilm formation, as well as the factors that influence biofilm formation on carrier materials, are discussed in Sect. "Biofilm formation", 'Biofilm Formation,' and Sect. "Carrier materials", 'Carrier Materials'). The carrier materials generally vary in size from 5–55 mm, with those having a pore size greater than 1 mm and spherical in shape are being selected based on specific treatment needs [7, 100]. Carrier materials helps to address the issue of biomass washout, ensures the development of a robust and diverse microbial community, which is essential for the AD performance and stability [7, 130]. For example, Pilarska et al. [131] utilized granular polylactide (PLA) as a carrier material during the anaerobic treatment of confectionery waste showed higher biofilm surface area (80%) compared to control group (40%). Zainab et al. [132] demonstrated significant enhancements in waste removal through the utilization of natural organic materials as biofilm carriers. The biofilm formed on a luffa sponge achieved 86% in VS reduction and 88% COD removal rates. This performance significantly outperformed the control, which only achieved a 51% reduction rate. Langer et al. [4] examined the dynamics of anaerobic biofilm attached on polypropylene carriers and observed significantly higher microbial count from biofilm. The total microbial count from the liquid and the biofilm was found to be 10^10^ and 10^11^ cells/mL, respectively. Furthermore, Show and Tay [133] found that support media with open-pored texture and high porosity facilitated the growth of a thicker and slimier biofilm and resulted in 77% COD removal rate compared to 57% removal rate achieved by support media with a smooth surface PVC and low porosity.

**Table 5:** Biofilm carrier material types and their application in AD

Carrier Material Type	Application/pollutant type	Reference(s)
Zeolites	Propionate degradation under ammonia stress, removal of ammonia	[134, 135]
Volcanic rock	Higher COD removal (up to 86–95%), higher organic removal (above 97%), higher methane yield	[136, 137]
Ceramics	Over 82–98% COD removal, high COD to VFA conversion rate, higher efficiency in biogas production	[137–139]
Activated carbon	High COD removal (up to 86%) at higher OLR, TS and VS removal, higher CH_4_ production	[138, 140, 141]
Polymeric biofilm carriers (*Kaldnes biocarrier, BioBall, blue media, Ultra Media, and Micro Media, polyethylene media*)	High TS, VS and COD removal (up to 90%), high methane production, enhance gas fermentation	[10, 137, 142, 161]
Glass based biocarrier	High COD removal (up to 86%), good for TS and VS removal	[141, 143]
Biochar	Higher and stable methane production, dissolved organic carbon removal, above 85% soluble COD removal, for tolerance against furfural and acetic acid	[144–146]
Straw based biocarrier	Higher COD removal, VFA removal, higher and stable methane yield	[143]
Luffa sponge	High COD removal (up to 95%), high methane concentration, above 85% VFA removal	[132, 137, 142]
Coconut husk fibre	Higher COD removal, up to 82% VS removal, TSS removal	[132, 147]
Woodchip	COD and VS removal, higher denitrification efficiency, higher methane yield	[132, 148, 149]
Bamboo carrier	Consistent and high COD and total COD removal, above 80% SS removal	[150, 151]

#### Formation on sludge granules

Anaerobic sludge granules (AnSG) represent another form of biofilm in anaerobic reactors. In AnSG, biofilm grows on sludge particles within sludge blankets acting as the carrier materials, where the microorganisms adhere to anaerobic flocs or clumps forming granular biofilms [152].

AnSG is characterized by its relatively higher organic removal rate, durability at high salinity, and enhancement of specific methanogenic activity [153]. AnSG can be applied to various reactor types, such as UASB, expanded granular sludge bed reactors, anaerobic sequencing batch reactors, and anaerobic baffled reactors [154–156]. Once a biofilm forms on the sludge particles, it improves the waste treatment efficiency and ensures a stable process, allowing the system withstands higher OLR, higher COD removal and achieving high methanogenic activity [157]. Wang et al. [158] reported that AnSG exhibited superior performance in total COD removal rates of 35.14% (470.8 mg/L) from high pH wastewater with high pH, compared to aerobic sludge granules. Sudmalis et al. [153] examined the role of AnSG in soluble COD removal at salinity levels of 5.0 and 20 g Na^+^/L. They reported that the soluble COD removal efficiency of the granules at 5.0 and 20 g Na^+^/L was found to be on average 96.9% and 93.4%, respectively. This higher removal efficiency in both salt concentration might be attributed to the formation of stable, fast, and robust granules.

In recent years, direct interspecies electron transfer (DIET) has been discovered as a form of extracellular electron transfer, enabling microorganisms exchange electrons to cooperatively degrade organic compounds under anaerobic conditions [165, 166]. In DIET, bacteria transfer electrons to other microorganisms (for instance methanogens) through electrically conductive pili and multihemec-type cytochrome, instead of interspecies H_2_/formate transfer. This mechanism allows methane production in a thermodynamically and metabolically more efficient manner [175]. Studies have shown that conductive biofilm carriers, such as granular activated carbon (GAC), carbon cloth, graphite, mediate DIET [167]. These carriers promote DIET-based biofilm, where electrons generated from electrochemically active bacteria, for instance *Geobacter* sp., can migrate through conductive carrier to methanogens [168]. Liu et al. [169] introduced novel graphite-modified carriers to AD, demonstrating these electron mediators suspended biofilm carrier significantly accelerated DIET and enhanced methane production [169]. Similarly, Zhang et al. [170] applied conductive biofilms in up-flow anaerobic sludge blanket (UASB), observing more effective performance over control.

### Norwegian case—full-scale biofilm plug-flow reactors (BPFR)

In general, full-scale biofilm based AD process have been widely reported, including fluidized bed reactor, sequencing batch biofilm reactor, and hybrid vertical anaerobic and aerobic biofilm reactor [171–173]. However, the application of these biofilm process has been limited to wastewater treatment, while there is limited information regarding the use of large-scale biofilm process for treating other substrates. A recent study from Yusof et al*.* [174] utilized pilot scale anaerobic biofilm digester to treat leachate from municipal solid waste (MSW). In addition, biofilm-based process for biological biogas upgrading has been able to scale up to industrial scales by companies such as Electrochaea, Biogasclean, or Q Power Oy. Application of biofilm plug-flow reactors for biogas production, especially at an industrial scale, is not as common as CSTRs. Here, a case study was present based on data obtained from biofilm plug-flow reactors (BPFR) in Norway.

**Fig. 2:**
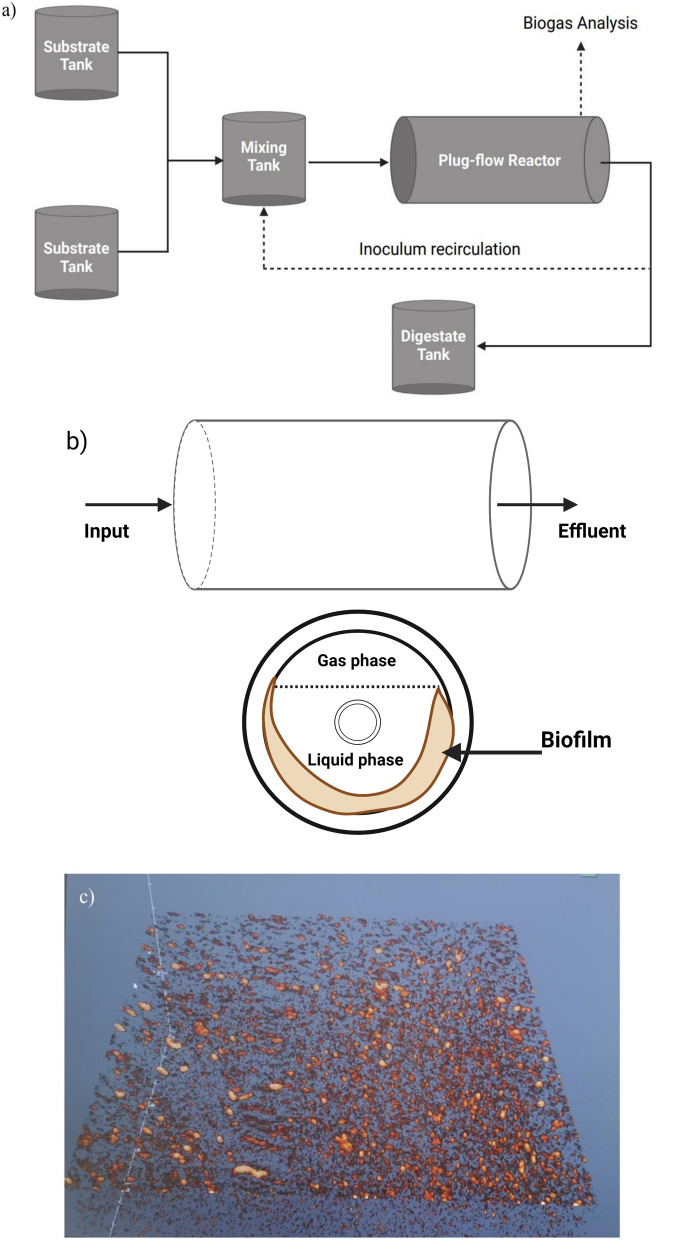
Biofilm plug-flow reactor (BPFR). **a** Schematic representation of the biofilm plug-flow reactor (BPFR) configuration. **b** Cross-sectional view of the BPFR showing the flow of liquid and gas phases through the reactor, with biofilm formation observed on the reactor walls within the liquid phase. **c** SEM image depicting spatially distributed biofilm formation on the surface of a material used to construct the interior of the BPFR wall (fiberglass). (Provided by Antec Biogas AS)

The data was based on two biogas plants with same reactor type: plant A, an industrial-scale plant, with a capacity of 10 × 10^5^ m^3^ and plant B, a farm-based biogas plant, with a reactor volume of 60 m^3^. The industrial-scale plant (Plant A) had an HRT of 14 days and was fed with cow manure (90–80%) and fish ensilage (10–20%). The farm-based 60 m^3^ reactor (Plant B) was fed with cow manure and operated with a 6-day HRT. The entire AD system consisted of a substrate tank, mixing tank, plug-flow reactor, and digestate tank (Figure 2**a**) BPFR combines plug-flow AD with biofilm to improve the uptake of organic matter by microbes (Fig. 2b). It is made up of (up to ten) chambers designed to distinguish distinct stages of AD, particularly acid generation and methanogenesis. The mixing system of the BPFR is operated by a shaft in the centre of the reactor, continuously and slowly rotating the chambers, pushing the substrates in a horizontal direction, allowing for radial mixing. Inoculum (digestate from the reactor) is recirculated to the mixing tank to enhance the anaerobic microbes, which could also initiate the hydrolysis process when different substrates are introduced.

An investigation of biofilm formation inside the reactor (BPFR) was performed. The biofilm developed on the reactor wall, which was composed of fiberglass. The spatial distribution of biofilm formation was investigated using optical coherence tomography technology (Ganymede™ Series SD-OCT system, Thorlabs GmbH, Lübeck, Germany), revealing heterogeneously distributed microbial islands of approximately 3 µm thickness at two different incubation times (Fig. 1c). Abundant microcolonies can be as effective as aerobic-like biofilms if optimum environmental conditions are maintained for attachment and growth, and higher detachment rates are avoided. A decrease in biogas production was observed when the detachment of microorganisms within the microcolonies increased [4], suggesting a higher abundance of methanogenic populations inhabiting the microcolonies. Advanced microscopy such as scanning electron microscopy and confocal laser scanning microscopy can be used to better characterize the biofilm populations. The BPFR demonstrated superior resilience to elevated acid concentrations (FOS to TAC ratio > 0.4), potentially attributed to phase separations and biofilm formation within the reactor. Moreover, data from Plant A suggested that the reactor could tolerate total ammonium concentrations of up to 5500 ppm.

## Microbial dynamics

In contrast to the planktonic lifestyle, the formation of biofilms results in alterations in microbial community structure, diversity, and biomass [159]. The microbial community structure within a biofilm is influenced by factors including the symbiotic or competitive relationship among microorganisms, the availability and composition of nutrients, and the resilience developed against environmental conditions.

Several studies have illustrated the microbial community dynamics within a biofilm. For example, Pilarska et al. [131] observed significant changes in the predominant microbial phyla, as the biofilm on the granular polylactide contained 35 more taxa; with *Firmicutes* (26.45%), and *Proteobacteria* (39.82%), while in the control the *Actinobacteria* was 34.87%, *Proteobacteria* (32.70%) and Firmicutes (19.45%). Langer et al. [4] found that biofilms during AD of sludge from meat industry had significantly higher microbial cell counts on polypropylene discs compared to reactor fluid. This highlights the intricate relationship between biofilm formation and microbial community structure changes.

Furthermore, Singh et al. [159] investigated microbial diversity within biofilms formed on various supporting surfaces. The researcher observed that the microbial community in the biofilm was more diverse than granules and planktonic microbes and *Methanosaeta* and *Cloacimonadaceae* thriving on granules and pumice stone supported biofilms, while *Pseudomonas* and *Acinetobacter* dominated the effluent community. Rademacher et al. [160] examined a two-phase biofilm-based reactor, revealing a distinct microbial structure between first stage (cellulolytic digester) and second stage (methanogenic digester). The archaeal population from the biofilm of the cellulolytic and methanogenic phases represents 2% and 12%, respectively. This might be connected to the development of a suitable environment for the archaea in the methanogenic phase, emphasizing how the dynamics and structure of the microbial population may be impacted by the stage of AD.

Various carrier materials may demonstrate different capacities for attachment. Liu et al. [161], for instance, identified polypropylene fibre (compared with polyester fiber, polyamide and fiberpolyurethane fiber) as an optimal carrier and significantly increased the abundance of key methanogenic genera, *Methanoregula*, *Methanosaeta* and *Methanobacterium* by 79.4%, 1.2%, and 18.3%, respectively. This implies the suitability of polypropylene fibres for the dominance of hydrogenotrophic and acetoclastic methanogens [162]. Thapa et al. [57] investigated the microbial dynamics during biomethanation of H_2_ and CO_2_ using a trickling filter bed reactor. They observed that *Firmicutes* was the predominant in the biofilm phase while *Proteobacteria* in the liquid phase. Similarly, the percentage of *Bacteroidetes* increased from 6.92% in the liquid phase to 19.6% in the biofilm phase. After the injection of H_2_ and CO_2_, the population of hydrogenotrophic methanogens increased. Specifically, the population of *Methanoculleus bourgensis* in the liquid and biofilm phases were 87.8% and 38.0%, respectively. Porté et al. [163] studied the microbial community structure in the biofilm and liquid phase in thermophilic trickling biofilter reactors for biogas upgrading. The researchers observed that *Methanothermobacter* spp. 1 (19%) and *Methanobacterium formicicum* (10%) dominated the biofilm phase whereas *Firmicutes* (40%) and *Proteobacteria* (22%) dominated the liquid phase. Tauber et al. [58] examined microbial community dynamics in a mesophilic biofilm reactor, revealing higher abundance of *Proteobacteria* and *Euryarchaeota* within the biofilm than in the liquid inoculum. The lower biofilm layer exhibited higher abundance, implying favourable anaerobic conditions for the proliferation of AD microbes. An increase in population counts and microbial diversity is associated with an environment rich in nutrients. This could not only reduce microbial wash but also promote cooperation and tolerance to inhibitors, leading to an increase in abundance and diversity within biofilms.

## Conclusion and perspectives

In this review, the current application of biofilm in AD is summarized with focus on impacting factors, optimization strategies, reactor types, and microbial communities. In general, biofilm significantly influences AD process as it improves the hydrolysis rate, enzymatic activities, mass transfer, and microbial diversity and resistance to inhibitors, thereby enhancing the efficiency and stability. The performance of biofilm is affected by parameters, including temperature, pH, competition/co-existence of microbes, shear stress, nutrients, carrier materials, and HRT. In recent years, there has been a trend in development of biofilm-based reactors with the goal of accelerating degradation rate or enhancing biogas upgrading. This presents new opportunities but also poses challenge. Future research is essential for the development of novel biofilm reactor processes or optimization strategies. In addition, there is a need to advance understanding of the functions and roles of microbes within biofilms.

## Data Availability

No datasets were generated or analysed during the current study.

## Abbreviations

AD: Anaerobic digestion
AnDMBR: Anaerobic dynamic membrane bioreactor
AnMBR: Anaerobic migrating blanket reactor
AnRBC: Anaerobic rotating biological contactor
AnSBR: Anaerobic sequencing batch reactor
AnSG: Anaerobic sludge granule
BOD: Biological oxygen demand
BPFR: Biofilm plug flow reactor
COD: Chemical oxygen demand
CSTR: Continuous stirred tank reactor
EPS: Extracellular polymeric substances
HRT: Hydraulic retention time
OLR: Organic loading rate
PLA: Polylactic acid
SCFA: Short chain fatty acid
SMX: Sulfamethoxazole
SRB: Sulphate reducing bacteria
SRT: Solid retention time
SS: Suspended solid
TAC: Total alkalinity concentration
TBR: Trickle bed reactor
TSS: Total suspended solid
TS: Total solid
UASB: Upflow anaerobic sludge blanket
VFA/FOS: Volatile fatty acid
VS: Volatile solid
VSS: Volatile suspended solid
